# Personal recovery, clinical recovery and patient-rated measures

**DOI:** 10.1192/j.eurpsy.2023.135

**Published:** 2023-07-19

**Authors:** M. Slade

**Affiliations:** School of Health Sciences, Institute of Mental Health, University of Nottingham, Nottingham, United Kingdom

## Abstract

**Abstract:**

This talk will cover two common areas of confusion. First, the relationship between personal recovery and clinical recovery will be described, using recent meta-analytic evidence. It will be argued that personal recovery is not the same as clinical recovery, and that there is now an established policy and practice consensus that supporting personal recovery is the primary aim of mental health systems. Traditional clinical recovery-oriented treatments which target for example symptomatology or relapse prevention can for many people with mental health issues contribute to their recovery at points in their lives, but for others different approaches are needed. This variation in clinical need is addressed in the second area – patient-rated measures. The rationale for measures of experiential knowledge will be given. A distinction will be drawn between Patient-rated outcome measures (PROMs) and Patient-rated experience measures (PREMs), and between peer-developed patient-generated PROMs (PG-PROMs) compared with those developed by non-peer research teams. It will be argued that modern mental health systems should be judged by their impact on recovery, as measured using PROMs and PREMs in preference to staff-rated measures.

**Disclosure of Interest:**

None Declared

